# Unpredictable Virulence of a Benign Commensal: Streptococcus salivarius Bacteremia and Infective Endocarditis in an Immunocompetent Patient

**DOI:** 10.7759/cureus.105713

**Published:** 2026-03-23

**Authors:** Erika J Bravo, Ahmed Gamal, Jennifer Kang, Kristina Maditz

**Affiliations:** 1 Internal Medicine, University Hospitals (UH) St. John Medical Center, Westlake, USA

**Keywords:** ceftriaxone, colonoscopy, dental procedure, infective endocarditis, meningitis, streptococcus salivarius

## Abstract

*Streptococcus salivarius* is a gram-positive viridans group streptococcus that primarily colonizes the oral cavity and gastrointestinal tract and is generally regarded as a benign commensal organism. Although invasive infections have been reported, they typically occur in immunocompromised hosts or following invasive gastrointestinal or spinal procedures. We present a rare case of *S. salivarius *bacteremia complicated by meningitis and infective endocarditis in an elderly immunocompetent patient following a routine dental cleaning.

A 74-year-old woman with a history of atrial fibrillation, hypertension, chronic obstructive pulmonary disease, and hypothyroidism presented with acute-onset severe headache, photophobia, nausea, and vomiting. Initial evaluation revealed leukocytosis and severe hypertension without focal neurological deficits. Within 24 hours, she developed acute encephalopathy. Blood cultures grew *S. salivarius*/*vestibularis *group. Cerebrospinal fluid analysis demonstrated marked neutrophilic pleocytosis and elevated protein, consistent with bacterial meningitis, though cultures were negative. Brain magnetic resonance imaging (MRI) raised concerns for purulent material, and computed tomography (CT) angiography revealed a small arterial communicating artery aneurysm. Transthoracic and transesophageal echocardiography identified mitral valve vegetation with moderate regurgitation, confirming infective endocarditis.

The patient was treated with intravenous ceftriaxone with significant clinical improvement and eventual resolution of the vegetation on follow-up imaging. This case is notable for the occurrence of *S. salivarius *bacteremia and infective endocarditis in an elderly, immunocompetent patient, emphasizing that this organism, though typically nonpathogenic, can cause severe invasive disease even in the absence of traditional risk factors.

## Introduction

Infective endocarditis has an incidence of approximately 2.6-7 cases per 100,000 persons annually in developed countries, with viridans group streptococci representing a major cause of community-acquired disease [1]. *Streptococcus salivarius* is a gram-positive commensal organism belonging to the viridans group that predominantly colonizes the human oral cavity, upper airways, and gastrointestinal (GI) tract [2]. Similar to most beneficial microbial communities inhabiting the human body, this organism plays a protective role against periodontal and GI illnesses through its anti-inflammatory properties [3].

However, *S. salivarius *can become pathogenic, causing bacteremia, intra-abdominal infection, pulmonary infection, infective endocarditis, meningitis, brain abscesses, and mycotic aneurysms under certain conditions [4-8]. The risk of developing these illnesses is increased in patients who are immunocompromised, those with underlying malignancy, or individuals who have undergone invasive spinal and GI procedures [8,9].

Although uncommon, *S. salivarius *has been reported as a causative organism of infective endocarditis within the viridans group streptococci. It was reported to have a moderate prevalence of infective endocarditis in patients with bloodstream infections, falling between low-risk species such as *Streptococcus pneumoniae *and very-high-risk species like *Streptococcus gallolyticus *[10]. In a study by Plainvert et al., *S. salivarius *accounted for two out of 58 (3.45%) cases of streptococcal infective endocarditis [11]. Similarly, in another study, *S. salivarius *group represented two out of 296 (1%) of the total cases of streptococcal infective endocarditis [12].

The patient in our case does not fit this typical risk profile. Despite her immunocompetent state, she developed infective endocarditis and meningitis in the setting of *S. salivarius *bacteremia after a recent dental cleaning [13,14].

## Case presentation

A 74-year-old woman with a past medical history of atrial fibrillation on rivaroxaban (with no valvular disease), hypertension, hypothyroidism, chronic obstructive pulmonary disease (COPD), and tubular adenoma of the colon presented to University Hospitals (UH) St. John Medical Center with several hours of sudden-onset severe generalized headache. The headache was accompanied by chills, photophobia, nausea, and vomiting. Symptoms occurred while driving home to Ohio from Pennsylvania after a family visit.

On initial presentation in the emergency department, the patient was afebrile (36.7°C) and severely hypertensive (225/94 mmHg) and had a heart rate of 60 beats per minute and an oxygen saturation of 98% in room air. She was alert and oriented, with a Glasgow Coma Scale score of 15. She did not have any focal deficit with a National Institutes of Health Stroke Scale score of 0; however, she exhibited a slow response. Physical examination revealed a supple neck without nuchal rigidity, regular rate and rhythm, and no murmurs. There were also no signs of infective endocarditis, such as Janeway lesions, Osler nodes, splinter hemorrhages, or Roth spots. A formal evaluation of the optic disc was not done; however, she did not have any complaints of blurring of vision or projectile vomiting.

Laboratory testing demonstrated leukocytosis of 16×10³/μL with neutrophilic predominance of 13.52×10³/μL (Table 1). Comprehensive metabolic panel (CMP), urinalysis, and troponins were unremarkable. COVID-19, Flu A/B, and respiratory syncytial virus (RSV) polymerase chain reaction (PCR) were negative. Computed tomography of the head and a chest X-ray showed no acute abnormality.

**Table 1:** Complete blood count with automated differential on initial presentation in the emergency department

Component	Results	Reference range
White blood cell count	16.0×10^3^/μL	4.4-11.3×10^3^/μL
Red blood cell count	4.84×10^6^/μL	4-5.2×10^6^/μL
Hemoglobin	12.9 g/dL	12-16 g/dL
Hematocrit	41.3%	36-46%
Platelets	258×10^3^/μL	150-450×10^3^/μL
Neutrophils	84.8%	40-80%
Automated immature granulocytes	0.4%	0-0.9%
Lymphocytes	8.7%	13-44%
Monocytes	4.4%	2-10%
Eosinophils	1%	0-6%
Basophils	0.7%	0-2%
Absolute neutrophils	13.52×10^3^/μL	1.6-5.5×10^3^/μL
Automated absolute immature granulocytes	0.07×10^3^/μL	0-0.5×10^3^/μL
Absolute lymphocytes	1.39×10^3^/μL	0.8-3×10^3^/μL
Absolute monocytes	0.71×10^3^/μL	0.05-0.80×10^3^/μL
Absolute eosinophils	0.16×10^3^/μL	0-0.4×10^3^/μL
Absolute basophils	0.11×10^3^/μL	0-0.10×10^3^/μL

She was admitted to the general medicine floor for hypertensive urgency with intractable headache and reactive leukocytosis of unknown etiology and was treated with empiric vancomycin dosed by pharmacy and cefepime 2 g every eight hours.

Within 24 hours of admission, she became lethargic and confused while remaining afebrile and hemodynamically stable. Blood cultures obtained in the emergency department grew *S. salivarius*/*vestibularis *group. Serum lactate increased from 2.5 to 4.6 mmol/L. Infectious disease and neurology consults were obtained due to concern for bacterial meningitis secondary to *S. salivarius *bacteremia. Cefepime was de-escalated to ceftriaxone 2 g intravenous (IV) q12h for improved central nervous system (CNS) penetration given concern for meningitis. Vancomycin continued pending final organism identification and susceptibility testing.

**Figure 1:**
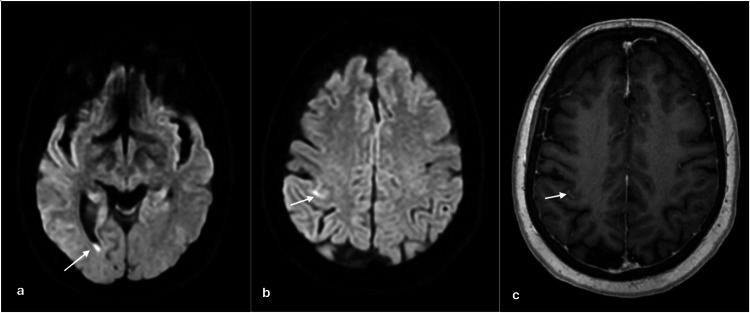
MRI of the brain with and without IV contrast revealing an abnormal diffusion restriction signal within the occipital horn of the right lateral ventricle (a), diffusion restriction signal abnormality within the sulci of the right cerebral hemisphere (b) with corresponding FLAIR hyperintensity, and abnormal enhancement (c) suggestive of purulent material MRI: magnetic resonance imaging; IV: intravenous; FLAIR: fluid-attenuated inversion recovery

Cerebrospinal fluid (CSF) analysis revealed leukocytosis of 5700/μL with neutrophilic predominance of 83%, elevated total protein of 243 mg/dL, and low-normal glucose of 46 mg/dL, consistent with bacterial meningitis. The meningitis pathogen panel and herpes simplex virus (HSV) 1 and 2 PCR were negative (Table 2). CSF gram stain did not show any microorganisms, and the microbial culture was negative. Non-contrast brain MRI showed small foci of diffusion-restricted material in the right cerebral hemisphere and right occipital horn with subtle enhancement, concerning purulent material collection (Figure 1). CT angiography of the head with and without IV contrast revealed a 5×4×3 mm saccular aneurysm of the anterior communicating artery.

**Table 2:** CSF analysis following lumbar puncture on day 2 of admission post-initiation of vancomycin and ceftriaxone CSF: cerebrospinal fluid

Component	Results	Reference range
CSF color	Straw	Colorless
CSF clarity	Cloudy	Clear
White blood cell count	5749/μL	1-5/μL
Red blood cell count	206/μL	0-5/μL
Manual neutrophils %	83%	0-5%
Manual lymphocytes %	7%	28-96%
Manual mono-/macrophages	6%	16-56%
Manual basophils	4%	Not established %
Protein	243 mg/dL	15-45 mg/dL
Glucose	46 mg/dL	40-70 mg/dL

**Figure 2:**
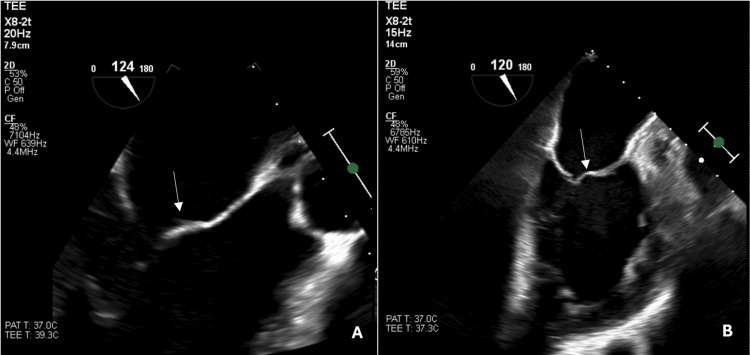
Transesophageal echocardiogram showing (A) a mobile linear vegetation on the anterior leaflet of the mitral valve and (B) the resolution of the mitral valve vegetation post-treatment

Given gram-positive bacteremia, a transthoracic echocardiogram was performed, which revealed a small mass with calcification at the base of the anterior mitral valve leaflet and moderate mitral valve regurgitation. Transesophageal echocardiography (TEE) confirmed mitral valve vegetation, establishing the diagnosis of infective endocarditis (Figure 2). Given MRI findings concerning for abscess formation as well as the TEE findings of small vegetation and moderate mitral valve regurgitation, she was transferred to University Hospitals (UH) Cleveland Medical Center for cardiothoracic surgery and neurosurgery evaluation.

At the tertiary center, she did not meet the criteria for urgent surgical interventions due to the absence of heart failure, conduction abnormalities, or significant neurologic deteriorations as well as the risks of surgery, which outweigh the potential benefit given other preexisting comorbidities. Antibiotic therapy was continued during her hospitalization, which led to improvement of her symptoms and mentation returning to baseline. She remained seizure-free during the entire hospital stay. Consequently, she was discharged home on a four-week course of IV ceftriaxone via a peripherally inserted central catheter.

At the outpatient follow-up, the patient reported only residual fatigue and mild headaches, presumed to be sequelae of meningitis. Two months later, repeat transthoracic echocardiography demonstrated the complete resolution of the vegetation with improvement in mitral valve regurgitation (Figure 2).

## Discussion

The source of bacteremia in this case is likely the non-invasive dental procedure one month prior to symptom onset, where transient bacteremia from gingival manipulation contributed to the development of infective endocarditis. Transient bacteremia is recognized to occur after dental scaling; however, observational studies indicate that the risk of infective endocarditis following dental cleaning is minimal and antibiotic prophylaxis is not advised in unselected cases [15,16].

Another potential contributor is the patient's colonoscopy with polypectomy three months prior to presentation, which has been reported to potentially increase the risk for bacteremia and, consequently, infective endocarditis secondary to mucosal trauma [16,17].

Despite negative CSF cultures, the CSF profile in this case was strongly suggestive of bacterial meningitis. Importantly, the patient received antibiotics 48 hours prior to sample collection, which can significantly reduce the CSF culture yield, as demonstrated in prior studies [18].

Ceftriaxone was selected as definitive therapy due to the organism's susceptibility profile. This resulted in complete resolution of the patient's mitral valve vegetation. While some *S. salivarius *strains demonstrate intermediate susceptibility/resistance to at least one beta-lactam antibiotic, ceftriaxone demonstrates activity against most strains, with only a 2.5% resistance rate [11]. Although penicillin susceptibility was present, ceftriaxone was favored for outpatient convenience given the prolonged treatment duration.

The 5×4×3 mm saccular aneurysm of the anterior communicating artery was unlikely to be mycotic in nature given its location. Mycotic aneurysms associated with infective endocarditis typically involve more distal cerebral vessels, most commonly the middle cerebral artery (55-77%), posterior cerebral artery distributions (18%), and less commonly the anterior cerebral artery. Therefore, no invasive neurosurgical intervention was pursued [16].

Previous reports have identified immunosuppression, malignancy, IV drug use, valvular disease, and invasive procedures as major risk factors for *S. salivarius *infection [4-9]. Uniquely in our case, the patient developed native infective endocarditis and meningitis despite being immunocompetent and lacking traditional risk factors. This case adds to the limited literature demonstrating that severe, life-threatening infections can occur in immunocompetent individuals [16].

## Conclusions

Although *S. salivarius *is a rare cause of infective endocarditis, the current case suggests a possible association between dental procedures and invasive infection with *S. salivarius*. It should also be considered as a potential pathogen capable of causing meningitis and infective endocarditis even in immunocompetent individuals. CSF analysis and echocardiography are critical diagnostic tools in such cases. Ceftriaxone demonstrates excellent activity against *S. salivarius *and represents an effective first-line treatment option.
